# Application of Online Anomaly Detection Using One-Class Classification to the Z24 Bridge

**DOI:** 10.3390/s24237866

**Published:** 2024-12-09

**Authors:** Amro Abdrabo

**Affiliations:** Department of Computer Science, ETH Zürich, 8092 Zurich, Switzerland; aabdrabo@ethz.ch

**Keywords:** structural health monitoring, online anomaly detection, supervised feature extraction, concept drift

## Abstract

The usage of anomaly detection is of critical importance to numerous domains, including structural health monitoring (SHM). In this study, we examine an online setting for damage detection in the Z24 bridge. We evaluate and compare the performance of the elliptic envelope, incremental one-class support vector classification, local outlier factor, half-space trees, and entropy-guided envelopes. Our findings demonstrate that XGBoost exhibits enhanced performance in identifying a limited set of significant features. Additionally, we present a novel approach to manage drift through the application of entropy measures to structural state instances. The study is the first to assess the applicability of one-class classification for anomaly detection on the short-term structural health data of the Z24 bridge.

## 1. Introduction

Anomaly detection methods have proven a remarkable success in various domains such as arrhythmia detection, firewall intrusion detection systems, credit card fraud, or detection of defects in mechanical parts [1,2,3,4]. However, all of these studies are distinct from structural health monitoring in terms of the availability of the labeled data. For example, for arrhythmia, data can be collected and monitored using smart watches, or for firewalls, where denial of service attacks can be simulated. One must hence take into consideration the scarcity of data for buildings or bridges, and in particular, labeled data regarding the structural state.

In recent times, the application of data-driven machine learning methods on vibration data, for the purpose of identifying damage in engineering structures, has seen a notable rise as shown by recent research [5,6,7,8]. As mechanical structures, bridges have natural frequencies that are determined by their physical properties such as stiffness and mass distribution. When subject to load, these structures vibrate at their natural frequencies. Due to the irreversible nature of most structural damage and the relatively recent development of remote sensing technology, accumulating data labels for every damage scenario proves to be prohibitively expensive. Simulations are therefore used to compensate for the lack of real-life damaged state training data. These simulations fail to capture some of the intricacies of sensor- based measurements, which include the noise in the sensor measurement, the difficulty of modeling boundary conditions, as well as the natural degradation of material. For a generic elastic mass system, the frequency and stiffness of the structure obey the FEM-based matrix equation $\left[\bar{K}\right]\lambda_{i}=\lambda_{i}\left[\bar{M}\right]\phi_{i}$, where $\bar{M}$ and $\bar{K}$ are the global mass and stiffness matrices, respectively [9]. The natural frequencies and modal shapes satisfy $\left[\omega \right]=\sqrt{\left[\lambda \right]}$ and $f=\frac{\omega }{2\pi }$. Therefore, a change in the natural frequency of a structure may indicate a change in the stiffness and consequently the health of the structure.

**Figure 1:**
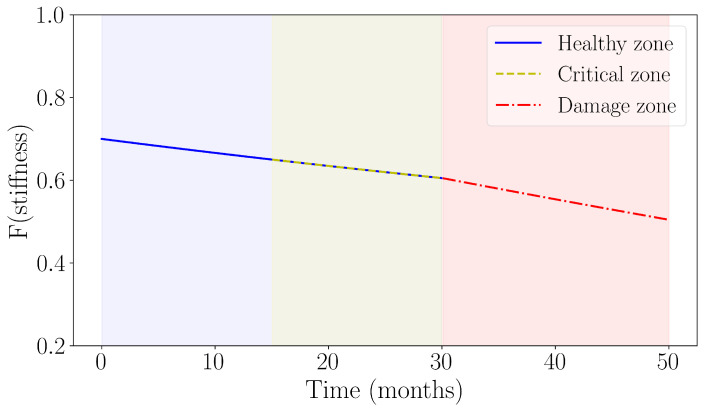
Reduction of a feature proportional to a stiffness that undergoes natural degradation. As stiffness is affected by damage, this is also a damage-sensitive feature (DSF).

To symbolically illustrate, Figure 1 only considers the evolution of a single damage sensitive feature. At the start of the measurement campaign, the bridge is healthy and its natural frequencies, along with the damage-sensitive feature, change slowly. As meteorological conditions change, the feature enters the critical state. As new disturbances are introduced, the distribution of the feature changes until eventually reaching the damage zone in red, which cannot be attributed to weather effects. The number of damage regimes is not known in advance for real-world data.

Online learning is a solution that tackles the lack of future knowledge of damage states by training on a small subset of the data. It has been shown to enhance structural health monitoring by strategically selecting data points for labeling/inspection [10,11]. It optimizes model training, reducing labeling efforts while improving accuracy. By prioritizing uncertain samples, active learning refines predictive capabilities, enabling a more efficient and reliable assessment of structural integrity and early detection of potential faults [11,12].

### Previous Work

Given the cost of obtaining labeled samples from buildings, numerous efforts have been concerted into using active-based learning techniques with a budgeted training sample size [5,11,12]. The class imbalance problem is a limitation in building monitoring, where there are a lack of monitoring data available for damaged buildings. A method for tackling this data availability problem is to examine data in a stream [5,13,14]. Hughes et al. define a methodology for structural health monitoring based on the expected utility, or risk, of inspection [5]. They define a cost and utility-based approach to SHM where health states are modeled using random variables in a probabilistic graphical model (PGM) [5]. Probabilistic graphical models are suited for a decision–theoretic approach under uncertainty wherein Bayesian networks and influence diagrams are used to model the expected gain in utility from having perfect information regarding the structural health state [5]. They further define an online training framework where points, which have large utility gain, are selected for labeling for use in a supervised Gaussian mixture classifier. A computational constraint of their approach is defining the transitional probabilities. In other words, it is required to have a posterior probability of the structural state based on the preceding structural state as well as the decision to maintain or not, which was taken during that preceding state.

This over-parameterized approach is alleviated by the usage of simple one-class classifiers as performed by [8]. Alternatively, it is also possible to use unsupervised clustering approaches [15]. However, no online framework for training is proposed in [8]. In this study, we aim to combine both the advantages of having an online training framework as well as simple and robust one-class classifiers. We also briefly examine the utility of finite-element models for assisting with the goal of structural health monitoring with particular application to relevant feature selection.

## 2. Z24 Short-Term Monitoring Campaign

The Z24 bridge was a classic post-tensioned concrete box-girder bridge with a 30 m main span and two 14 m side spans [16]. The bridge was demolished in 1998 and was monitored under the European Brite EuRam research project BE-3157 [16].

**Figure 2:**
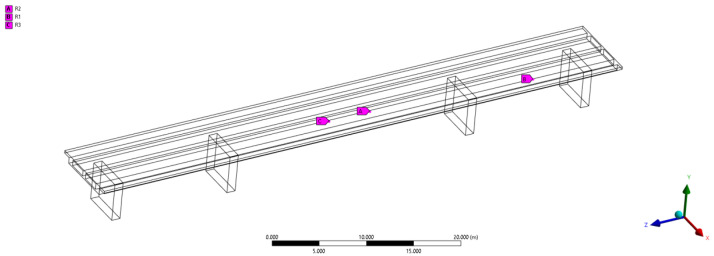
Wireframe of the Z24 bridge on ANSYS 2023 R1. The Z-axis defines the longitudinal direction. The Y-axis defines vertical, and the X-axis is transversal. Sensor R1 is at location B. Sensor R2 is at A, and sensor R3 isat C. Only these sensors are used, as they were the only sensors which were not moved during the short-term monitoring campaign.

The dynamics of the bridge were monitored by measuring 16 accelerations at various points and directions on the bridge [16]. After each hour, a setup of sensor locations was used to measure environmental data using 48 sensors along with 65,536 acceleration samples from 16 acceleration sensors operating at 100 Hz, which was repeated for ten setups [16]. However, in this study, only sensors R1, R2, and R3 were used, given that their position did not change from one setup to the next. The approximate locations of these sensors are shown with purple tags in Figure 2. For the case of R1, the acceleration was measured in the vertical direction, whereas for R2, the acceleration was measured in the longitudinal, vertical, and transversal direction. For R3, only the vertical acceleration was measured. The 17 damage scenarios, as well as the period in which they were conducted, are shown in Table 1.

## 3. Data Preprocessing

The ambient excitation files are collected, and only measurements relating to sensors R1V, R2L, R2T, R2V, R3V are kept. As not all sensors acquisitions had the same length, each column is padded using the starting rows with the necessary length to achieve a length of 589,824 rows. For each damage scenario, a dataframe, from the concatenation of the used channels, is computed, which is vertically divided into windows with 16,384 samples, thereby yielding 36 observations of dimension (16384, 5) for each damage scenario. In total, 544 observations of the aforementioned dimension were used for training, and 68 were used for testing, thereby defining a train–test split of 90–10% for use in supervised feature selection.

### 3.1. Frequency Domain Visualization

**Figure 3:**
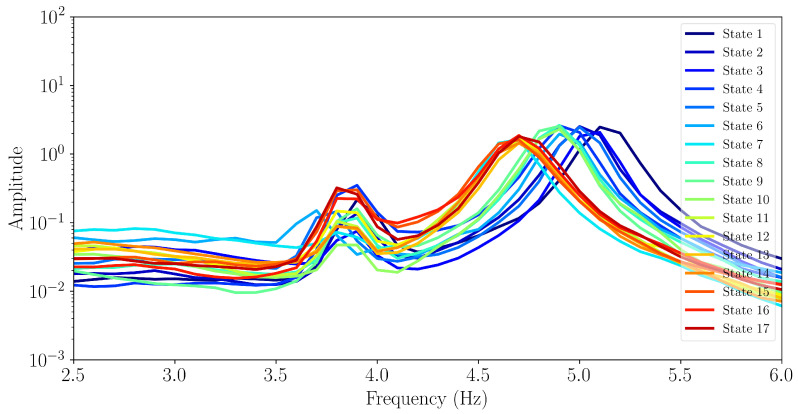
Reduction in natural frequency of second mode (near 5 Hz), from the power spectral density along R2T, visible due to damage progression.

The power spectral density (PSD) is given by $S_{x}\left(e^{j\omega }\right)={\left|X\left(e^{jw}\right)\right|}^{2}$, where $X(e^{jw})$ is the Fourier transform of x[n] the acceleration signal. We use Welch’s method to compute it using a segment length of 1000. In the case of the R2T and R1V channel, the PSD is shown in Figure 3, where the gradual reduction in frequency is observable.

### 3.2. Damage-Sensitive Features

The usage of damage-sensitive features in SHM is necessary for the early detection of structural damage and the optimal forecasting of maintenance operations. These features enable a shift toward predictive maintenance models, which enable the efficient use of sensor data while reducing maintenance-induced downtime. The results of DSF extraction from the detrended acceleration signal are shown in Table 2 [17].

These features were computed for each channel, yielding 240 features. The descriptive statistics include the mean, median, variance, kurtosis, skew, and mode. The lengths and repetitions refer to the number of zeros or NaNs, longest streaks above mean, number of peaks in lag period of at most 3000 samples, and similar counts. Statistical combinations comprehend the root mean square of the signal, the mean of the absolute value of changes in adjacent time-series values, as well as the mean value of the central approximation of the second derivative among other computations. Instead of being computed separately on each channel as the previous features, the transmittance is defined as $T\left(f\right)=\frac{|PSD_{i}\left(f\right)|}{|PSD_{j}\left(f\right)|}$ where *i* and *j* are different channels [18], with *j* being the reference channel, in this case R1V. The features computed for the transmittance and the power spectral density are listed in Table 3.

Afterwards, a standardization operation is applied with mean imputation for columns that are at least 90% complete, resulting in a training data of shape (544, 906) and testing data of shape (68, 906).

### 3.3. Feature Visualization

**Figure 4:**
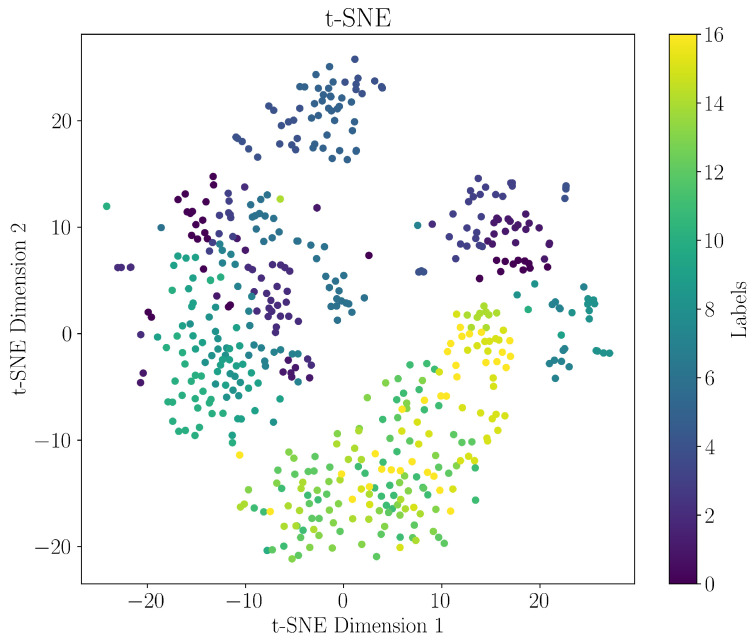
A t-SNE visualization on the training set. Labels correspond to the state from Table 1.

For the visualization of high-dimensional spaces in low dimensions, a common approach is t-stochastic neighbor embedding (t-SNE). This neighbor-embedding algorithm transforms the similarities among data points into joint probabilities using a Gaussian kernel in the original feature space and a Cauchy distribution in the reduced space [19]. As such, it aims to preserve the local structure. The result is shown in Figure 4.

**Figure 5:**
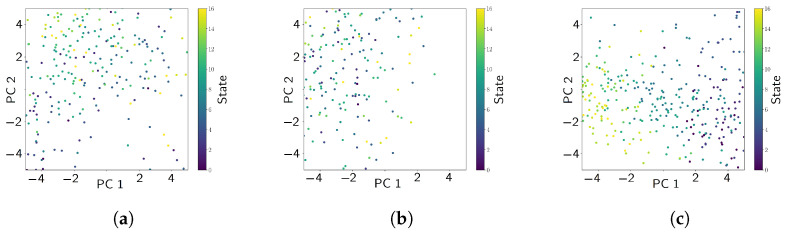
PCA applied over different feature subsets. (**a**) Features from Table 2 applied to all channels (R1V, R2L, R2V, R2T, R3V). (**b**) Features from the PSD of the channels. (**c**) Features derived from the transmittance with respect to R1V of the remaining channels.

Another technique for dimension reduction that is more stable than neighbor embedding, due to its lack of dependence on randomized gradient descent, is principal component analysis. Before any supervised models were applied, the features derived from transmittance produced the most distinguishable state-based deviations based on the projection, as depicted in Figure 5c. We can thus expect to find that most of the important features are from the transmittance.

## 4. Feature Selection and Dimension Reduction

A limitation of unsupervised methods in general is their reliance on the distance metric. With 906 features involved, pair-wise distances are meaningless due to the dimensionality curse. Thus, we use supervised techniques for feature selection. For fully supervised training, three methods were tested: XGBoost, SVC, and KNN. The grid for each is listed in Appendix A.1. Before each, a feature importance experiment was performed to reduce the dimensions of the data and prevent overfitting. For KNN, a PCA step was additionally performed on the features to help stabilize the training of the algorithm by preventing neighboring points from being farther apart.

### 4.1. Supervised Feature Selection Methodology

**Figure 6:**
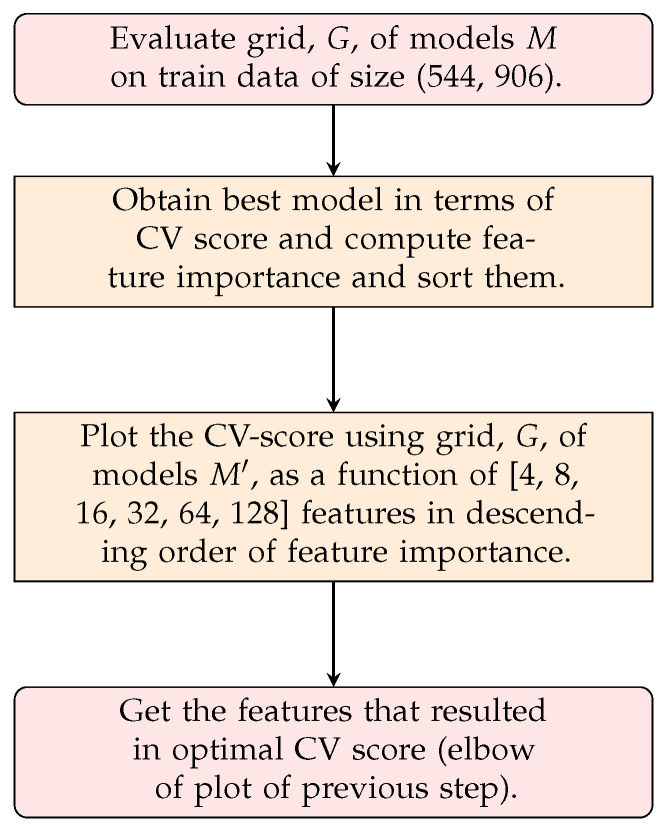
Supervised feature selection framework. Models *M* are used for feature selection, and $M^{\prime }$ are used for assessing the quality of found features. Here, *M* can be KNN, SVC, or XGBoost, where $M^{\prime }$ is correspondingly relief feature selection, logistic regression, and XGBoost, respectively.

The methodology for supervised feature selection is shown in Figure 6. Note that this methodology does not necessarily produce the features that work best for *m* features, where *m*<< 906. This is due to the fact that the features are ordered according to the evaluation of the model, M, on the entirety of the feature space. Hence, if a model uses distance-based metrics to form its decision space, then it will still suffer as a result of using all features.

### 4.2. XGBoost

The feature importance in gradient boosting is calculated in the same manner as in decision trees. However, the importance is averaged across all the trees. Assuming node *i* of a tree is split using feature *f*, the feature importance for feature *f* at node *i* is given by Equation (1) [20]. Note that this is only for one node. For a tree, this is summed across all nodes, and for an ensemble of trees, as in gradient boosting, this is further summed across all trees. Here, we denote $left_{i}$ and $right_{i}$ as the nodes to the left and right of *i*, respectively. NI is the node impurity, which provides a measure of how uniformly the node splits data points.

(1) $${\mathrm{Importance}}_{f,i}=NI_{i}\times \frac{N_{i}}{N_{\mathrm{total}}}-NI_{left_{i}}\times \frac{N_{left_{i}}}{N_{\mathrm{total}}}-NI_{right_{i}}\times \frac{N_{right_{i}}}{N_{\mathrm{total}}}$$

The windows are zero-indexed. The features extracted from XGBoost, in order, are the relative position of the maximum within the fifth window of size 2.5 Hz obtained by dividing the interval of [0, 30 Hz] into contiguous windows of size 2.5 Hz. The second is the range of frequencies which contains 50% of the sum of values of the amplitudes of the Fourier transform of the signal along the R2V channel. The third is the standard deviation of the prominence of the peaks for the transmittance from the R2T channel. The last feature is the relative position of the maximum in the second window (of size 2.5 Hz) of the transmittance of the signal along the R2L channel.

### 4.3. Support Vector Classification

Given the strong effect of the geometry of the points in a dataset on the output of support vector classification, we rely on logistic regression to compute the importance of features. The objective of the support vector involves the dot product of a weight vector with an instance, which is similar to that of logistic regression. Additionally, to prioritize spares weights, we use for regularization the L1 loss.

**Figure 7:**
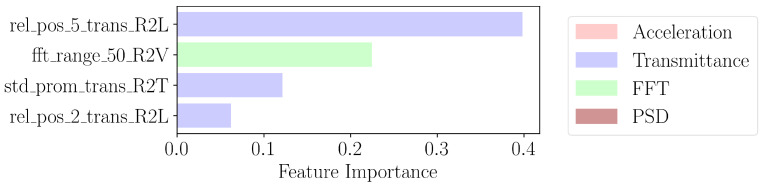
Four most important features extracted by XGBoost in third step of Figure 6.

**Figure 8:**
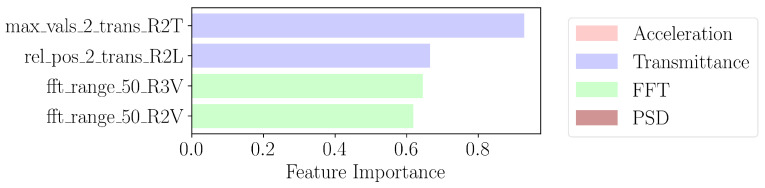
Four most important features extracted by logistic regression in third step of Figure 6.

In contrast to the importance extracted by XGBoost in Figure 7, the optimal found number of features in logistic regression is higher at 32, indicating worse compression than XGBoost. This limitation can be explained due to the linear relation assumption of logistic regression. Between the ranking shown in Figure 7 and Figure 8, we notice similarities in the top four features. In particular, the rel_pos_2_trans_R2L feature signifies the relative position of the peak of the transmittance along R2L within the second window containing the first mode. Another shared characteristic is the fft_range_50_R2V feature, which is the frequency bandwidth containing the total sum of amplitudes of the Fourier transform along the R2V channel. The most important feature, max_vals_2_trans_R2T, which denotes the maximum value of the transmittance in the second window along R2T, is not scale-invariant, unlike the relative position, as multiplying the signal by a factor changes the feature. Note that here, step 1 of Figure 6 uses logistic regression to order the features, but the later steps use SVC to avoid the linear relation assumption.

### 4.4. K-Nearest Neighbors

**Figure 9:**
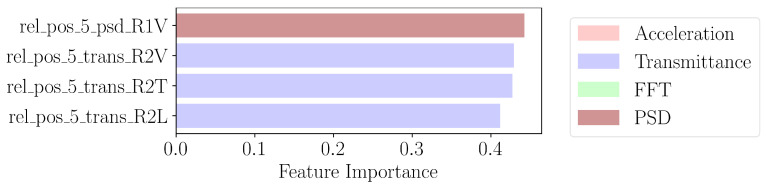
Four most important features extracted by Relief in third step of Figure 6.

As nearest neighbors do not provide feature selection, we use the ReliefF algorithm [21]. The algorithm is a modification of the original Relief algorithm [22], which proposes updating the *i*th coefficient of the importance weight vector, *W*, as in Equation (2). The nearest miss, $M_{i}$, is the closest instance of a different class than the randomly selected instance whose feature vector is denoted $x_{i}$. Likewise, the nearest hit, $H_{i}$, is the closest instance of the same class. The ReliefF algorithm proposes several updates. First, it averages over the *k* nearest misses and hits. It also performs the update for as many instances as available. Before the grid of KNN models is trained, we first apply PCA with the number of components in the range [3, 6, 12] on the training data where only the top features, in the range [4, 8, ⋯, 128], found from ReliefF are kept. The ranked importance of the features is shown in Figure 9.

(2) $$W_{i}=W_{i}-{\left(x_{i}-H_{i}\right)}^{2}+{\left(x_{i}-M_{i}\right)}^{2}$$

### 4.5. Summary and Comparison

The three methods, KNN, SVC, and XGBoost, demonstrated considerable overlap in the most important damage-sensitive features retrieved. In all cases, the importance of the transmittance is emphasized, in particular, the relative position of the maximum within the fifth 2.5 Hz bin.

**Figure 10:**
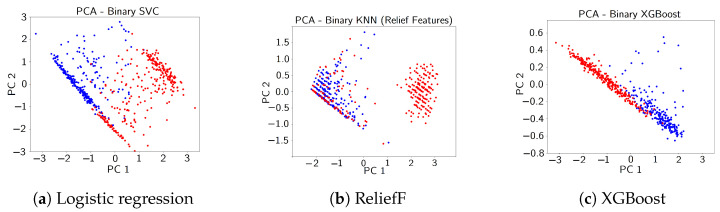
PCA of top four features of ReliefF from Figure 9, XGBoost from Figure 7, and logistic regression from Figure 8. Damaged state points are in red, while healthy state points are in blue.

**Figure 11:**
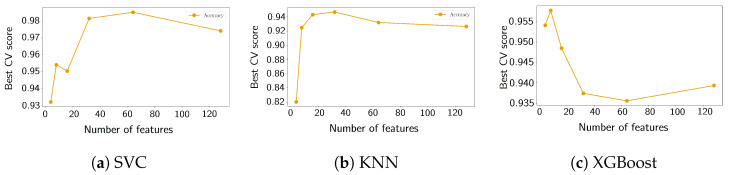
CV-scores obtained from step 3 of Figure 6 for supervised classification methods. XGBoost achieves the highest CV-score for 4 features at 0.95.

The two-dimensional PCA projections of the training data, filtered by the found features, are shown for each method in Figure 10. The higher the number of features, the more data have to be accumulated to derive a meaningful PCA projection. The separation achieved by the four features identified through XGBoost, shown in Figure 10c, outperforms that of features derived from other methods. Moreover, it maintains relatively high cross-validation score accuracy, as demonstrated in Figure 11, making it the preferred choice for our online anomaly detection. Additionally, XGBoost gave the highest test accuracy using the top 4 features: 0.94 compared to 0.94 for SVC and 0.82 for KNN. SVC achieved a higher cross-validation accuracy (0.98) while using far more features than XGBoost and having the same test accuracy. Hence, XGBoost has a lower variance compared to the SVC model.

## 5. Online Anomaly Detection

**Figure 12:**
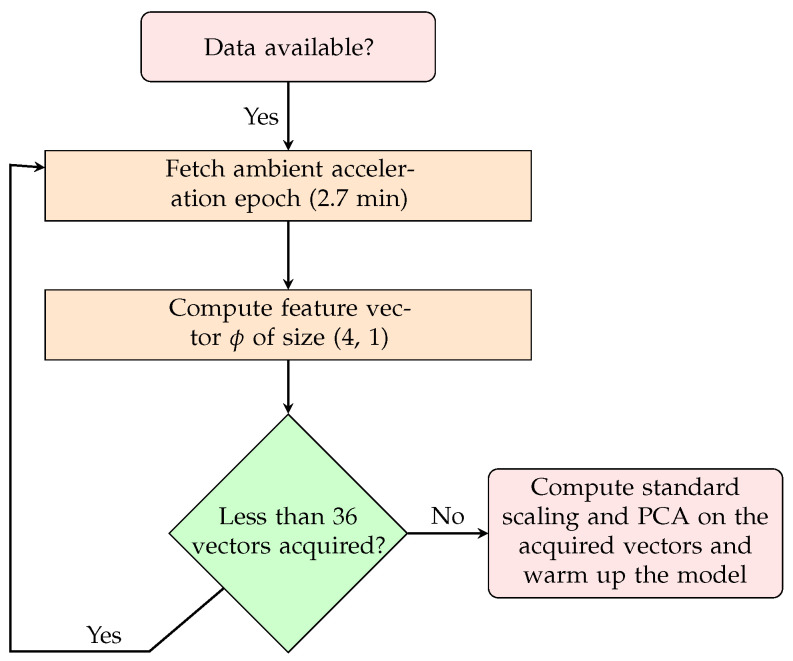
Standardization and PCA on data from first state.

Given the limited amount of data covered in the Z24 short-term dataset and that most damage cases were as a result of disrupting the tendons acting in the longitudinal direction, it is possible to derive a small number of features to obtain meaningful results. As such, we use the features from XGBoost from Figure 7. To prevent overfitting and overcome the dimensionality curse, the original four features, represented as ϕ, are compressed to three variables, represented as ψ, using principal component analysis. To maintain practical applicability, standard scaling and PCA over the aforementioned features are not computed over the entire manifold of healthy instances. Instead, they are only computed on the first health scenario, which comprises the first 36 instances of the dataset. The process is depicted in Figure 12.

**Figure 13:**
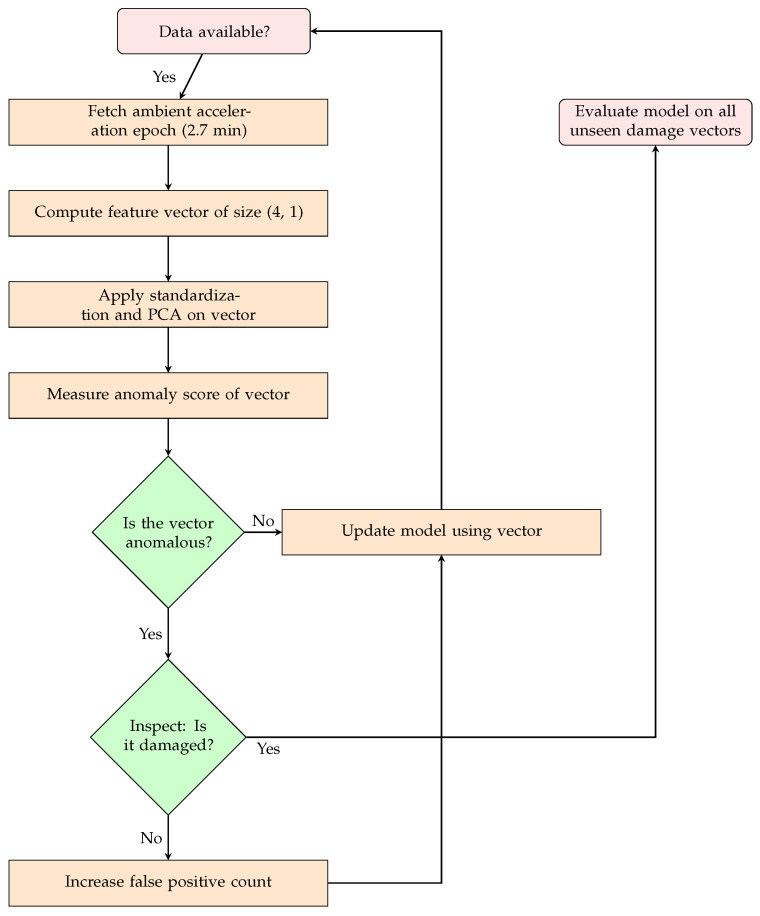
After PCA and standardization have been initialized in Figure 12, the OCC model is trained using the procedure shown here. For cross-validation, the performance on the omitted healthy scenario is evaluated instead of the unseen damage set.

After the first data points from the first health scenario are collected to initialize PCA and standard scaling, the model is then pretrained on these instances, and the remaining healthy scenarios from the second to the last healthy scenario are used as the healthy data stream for training and/or cross-validation. Cross-validation is evaluated by removing a healthy scenario, except for the first scenario, running the loop shown in Figure 13 with the sole modification being that the model accuracy is evaluated on the unseen/removed healthy scenario. After the optimal hyperparameters are found, we run the loop again using all healthy scenarios and evaluate on the unseen damaged set of data as in Figure 13. The grid for the hyperparameters is listed in Appendix A.2.

To replicate scenarios where damaged data are unseen for most of the measurement campaign, the test set consists only of the damage scenarios. We chose having only damage cases in the test set, as including healthy data into the test set would actually increase the performance of the model on the damage data, since the predictive model would have a smaller support volume due to the reduction in updates. However, to test the robustness of the models compared, the training loop does not stop when the model no longer receives updates from instances from the healthy training set. Instead, it continues being updated with damaged instances until the model finally detects an anomaly.

### 5.1. Elliptic Envelope

Worden et al. demonstrated the use of the Mahalanobis distance for outlier analysis in several case studies including a gearbox [7]. In order to adapt to realistic scenarios, the support should be implemented incrementally and using constant memory. Hence, naively storing all arriving instances is not allowed. The simplest approach is using an elliptic envelope, characterized by Gaussian mean and covariance matrix, as shown in Equation (3).

(3) $$f\left(\psi \right)=N\left(\mu ,\Sigma \right)=\frac{1}{{\left(2\pi \right)}^{3/2}{\left|\Sigma \right|}^{1/2}}\exp\left\{-\frac{1}{2}\left(\psi -\mu \right)⊤\Sigma^{-1}\left(\psi -\mu \right)\right\}$$

Update rules for the elliptic envelope are succinctly described by the running statistics in Equations (4) and (5), which are the maximum-likelihood estimators for the mean and covariance, respectively [23].

(4) $$\mu_{i}=\mu_{i-1}+\frac{\psi_{i}-\mu_{i-1}}{i},i\geq 1,\mu_{0}=0$$

(5) $$\Sigma_{\psi ,i}=\frac{\left(i-1\right)\times \Sigma_{\psi ,i-1}+\left(\psi_{i}-\mu_{i}\right){\left(\psi_{i}-\mu_{i}\right)}^{T}}{i},i\geq 1$$

An anomaly occurs if a point’s Mahalanobis distance to the envelope’s mean exceeds the threshold defined by the 0.95 confidence level. As this corresponds to a significance level of 0.05, this implies a 5 percent chance of incorrectly identifying a healthy domain point as anomalous. The prediction rule is translated mathematically by Equations (6) and (7), with the latter based on an approximation that the Mahalanobis distance for a multivariate Gaussian variable closely follows a Chi-squared distribution [6].

(6) $$\begin{array}{cc} D_{i}^{2} & ={\left(\psi_{i}-\mu_{i-1}\right)}^{⊤}\Sigma_{i-1}\left(\psi_{i}-\mu_{i-1}\right) \end{array}$$

(7) $$\begin{array}{cc} D_{i}^{2} & >\chi_{0.95,3}^{2} \end{array}$$

Once the condition in Equation (7) is fulfilled, an anomaly is triggered. The 0.95 threshold in the Chi-squared distribution is fixed before cross-validation, since cross-validation automatically chooses the highest threshold to minimize false positives. The Monte-Carlo method can be used to calibrate the threshold as demonstrated in [7]; however, in this study, we apply the common two standard deviation rule. The strength of this method lies within its simple parametrization, which inherently acts as a form of regularization, as well as the simple ellipsoid-shaped decision boundary and small memory complexity. The downside is the strong assumption that the decision boundary is ellipsoid; in other words, the support of healthy samples follows a Gaussian distribution. Another disadvantage is that updates that occur for healthy outliers yield more change to the shape of the ellipsoid than normal healthy points.

### 5.2. Incremental One-Class Support Vector Classifier

One-class SVC is a variant of SVC that aims to find a hyperplane that separates regions with a high concentration from regions with low concentration of instances [24]. The objective is given by Equation (8), subject to Equation (9). The algorithm for updating the model is listed in Algorithm 1.

(8) $$\min_{w,\xi ,\rho }\frac{1}{2}{∥w∥}^{2}+\frac{1}{\nu n}\sum_{i=1}^{n}\xi_{i}-\rho$$

(9) $$w\cdot g\left(\psi_{i}\right)\geq \rho -\xi_{i},\;\xi_{i}\geq 0,\;\forall i$$

**Algorithm 1:** Update Method of Incremental OC-SVC **Input**: SHM vector ψ **Data**: Buffer *B* of capacity *c*, pretrained model *S*, retrain_size *R* 
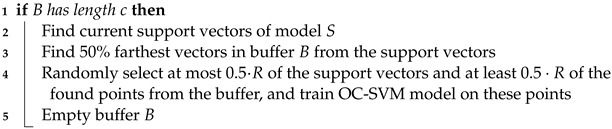


Note the pretraining referred to in Algorithm 1 is performed once and is the pretraining referred to in Figure 12. The prediction is simply given by the prediction of the SVC model, as in Equation (10), where *k* denotes the kernel transformation used, and the $\alpha_{i}$ values are found using quadratic programming.

(10) $$f\left(\psi \right)=\mathrm{sgn}\left(\sum_{i}\alpha_{i}k\left(\psi_{i},\psi \right)-\rho \right)$$

The advantage presented by OC-SVC, over the elliptic envelope, is the ability to find a region that is more complex regarding the function of its kernel. In this case, the RBF kernel $k\left(x,x^{\prime }\right)=g\left(x\right)\cdot g\left(x^{\prime }\right)=\exp\left(-\frac{||x-x^{\prime }{\left|\right|}^{2}}{2\sigma^{2}}\right)$ is useful as it results in an SVC model which has nearest-neighbor properties. In particular, this signifies robustness to non-linear separability and greater adaptability to multimodal distribution among the healthy instances.

The incremental version of OC-SVC implemented here defines a retraining parameter and a buffer size. As points arrive to the model’s update procedure, as shown in Figure 13, they are accumulated into a buffer. Once the buffer is full, the model is updated. The update is performed by training on a parameterized number of points. Approximately half of these points are from the model’s current support vectors, and the other half come from the points in the buffer that are farthest, in Euclidean space, from the aforementioned support vectors. Once the update is completed, the buffer is emptied and ready to receive points again.

To reduce sudden changes in the SVC parameters, one can implement the buffer in queue fashion, removing only the oldest point in the queue on each update call to the model. Likewise, one can update SVC using the closest (not farthest) 50% of points to the current model’s support vectors. However, in this study, we chose to have a model which adapts faster to changes in the concept of healthy instances to reduce the number of false positives. The clear disadvantage of this approach is the proneness to steep changes in the decision boundary caused by the buffer regularly emptying as well as the sensitivity to the parameters controlling the number of points for retraining as well as buffer size.

### 5.3. Half-Space Trees

**Figure 14:**
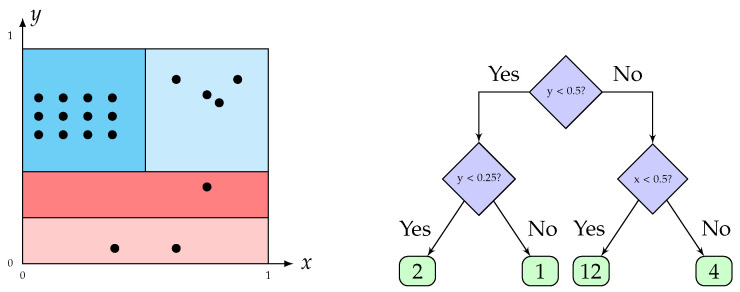
Example of a half-space tree for two-dimensional data. Red and blue regions correspond to anomalous and non-anomalous regions, respectively.

As the name suggests, half-space trees (HSTs) are trees where each node divides the feature space into two subspaces. The selection for which dimension to split is random. The logic behind HST is that each node maintains a count, called mass, of how many training instances are observed within the subspace of that node [25]. Two mass profiles are maintained at each step of the stream: a mass profile used for anomaly detection and a mass profile that is being constructed based on the instances arriving in the current window. Hence, a mass profile is constructed during training, where the mass at a node corresponds to the number of instances in the subspace defined by the node. Instances that arrive in nodes with low mass are given high anomaly scores. To compute the anomaly score of an instance, for each tree *T*, one traverses the tree *T* from the root to a node *k*, located at depth d[k], which contains m[k] instances. This is intuitively depicted in Figure 14. The exact formula for computing the anomaly score of an instance is thus given by Equation (11).

(11) $$\sum_{T\in \mathrm{Trees}}m_{T}\left[k\right]\cdot 2^{d_{T}\left[k\right]}$$

In this work, we use River 0.19.0, which computes anomaly scores between 0 and 1. As such, we fix a threshold of 0.7, above which an instance is classified as anomalous. As the model establishes its mass profiles based on a small window of data, HSTs work well when anomalies are spread out and poorly if the anomalies arrive together in small windows, as the HSTs would be fooled into expecting high mass due the high rate of arrival of anomalous observations. Another drawback of HSTs is that in order to compute equal-volume half-spaces, one needs all features to be min–max scaled to be in the interval [0,1]. Min–max is achieved in an online streaming fashion with the use of the River package. However, since the mass profile, which is used to determine anomaly scores, does not transfer its masses or beliefs to the mass profile being constructed, half-space trees adapt fast to concept drifts and multimodal data. Similar to the elliptic envelope, this method features constant amortized memory and runtime [25].

In this study, a depth of 3 or 4 is fixed for the trees, corresponding to the fact that one would like to classify all healthy points into one large octant. Of course, the decision boundary will not exactly resemble an octant given that one averages the scores of multiple trees to obtain a final anomaly score.

### 5.4. Local Outlier Factor

Local outlier factor (LOF) works by comparing the reachability density of a point to that of its neighbors [26]. If a point has high or similar density compared to its neighbors, its LOF score will be less than or equal to 1 and hence an inlier. Otherwise, it is considered an outlier. LOFs, despite being designed for anomaly detection in static pool cases, can be adapted to work on windows similar to HST. Unlike the OC-SVC method, where the buffer is emptied upon retraining, for LOFs, the buffer is implemented in a queue-like fashion to avoid sudden changes in the computed local reachability densities of the points in the buffer. To maintain realistic applicability, all buffer sizes are limited to a maximum size of 36. Each update follows Equations (12) and (13), and the prediction method follows Equation (14). Note the exact implementation for the LOF score is based on sklearn 1.3.0, which shifts and offsets the score to have inliers positive and outliers negative.

(12) $${\mathrm{reachability}-\mathrm{distance}}_{k}\left(A,B\right)=\max\left\{d\left(A,B\right),k-\mathrm{distance}\left(B\right)\right\}$$

(13) $${\mathrm{LRD}}_{k}\left(A\right):=\frac{1}{\frac{1}{|N_{k}\left(A\right)|}\sum_{B\in N_{k}\left(A\right)}{\mathrm{reachability}-\mathrm{distance}}_{k}\left(A,B\right)}$$

(14) $${\mathrm{LOF}}_{k}\left(A\right)=\frac{\sum_{B\in N_{k}\left(A\right)}{\mathrm{lrd}}_{k}\left(B\right)}{|N_{k}\left(A\right)|\cdot {\mathrm{lrd}}_{k}\left(A\right)}$$

k−distance(B) is the distance from point *B* to its *k*th nearest neighbor, and $N_{k}\left(A\right)$ defines the set of *k* nearest neighbors of *A*. In this study, the Euclidean distance is used to define d(A,B). Note that this method is very susceptible to the choice of *k* and the size of the window. If both parameters were to be similar, then the LRD of all points would likewise be similar, and consequently, the confidence in the LOF scores would diminish. Similarly, if *k* is set too low, given that it has to be smaller than the window size, then we risk increasing the false positive rate and hence inspection cost. Here, the two hyperparameters are the neighbor count and the queue size for the calls to update.

### 5.5. Entropy-Guided Elliptic Envelopes

Entropy-guided elliptic envelopes (EGs) are an attempt to enhance the elliptic envelopes approach by having a new envelope formed when an incoming point, or batch of points, have a high entropy score, where entropy is calculated using Equation (16). The use of entropy has already shown promising results in the context of active learning for sample selection [13]. If we were to reset the predictive models when an anomaly occurs, each reset would incur the cost of reducing the confidence in predictions as well as the cost of higher rate of false positives. As such, resetting the model on each anomaly may not be optimal.

We begin by showing how the entropy is calculated. First, for logistic regression, the probabilities are defined as in Equation (15), where $C_{1}$ refers to the healthy class [27].

(15) $$p\left(C_{1}|\psi \right)=y\left(\psi \right)=\frac{1}{1+e^{-w^{T}\psi }}$$

The likelihood, or the probability of the true labels given the weight, is maximized using gradient descent. Thus, the weight vector *w* is found in an iterative fashion. The entropy is given by Equation (16). The probabilities and consequently the entropy depend on the logistic regression model.

(16) $$H\left(\psi \right)=-\left(1-p\left(C_{1}|\psi \right)\right)\log_{2}\left(1-p\left(C_{1}|\psi \right)\right)-p\left(C_{1}|\psi \right)\log_{2}\left(p\left(C_{1}|\psi \right)\right)$$

For this approach, the model is reset once it detects an entropy surge from the output of the incremental logistic regression. The intuition lies in the fact that points from similar distribution will follow patterns of decreasing entropy, whereas a sudden jump in entropy indicates a deviation from the distribution. Note that in the early stages of training the model, the probabilities, assuming a randomly initialized *w*, are on average close to half, which leads to high values of the entropy in Equation (16). Assuming linear separability, all the instances will converge, in terms of probability in Equation (15), toward their correct labels. For this study, the data are not linearly separable; however, the entropy still presents useful information as a measure of the anomaly of an instance. The update logic is shown in Algorithm 2, where the cross-validation hyperparameters are the regularization factor (L2) for the logistic regression model, the window size *W*, and the significance factor *s*. For prediction, we simply use the most recent Gaussian envelope to make predictions (see prediction in Section 5.1).

**Algorithm 2:** Update Method of Entropy-guided Elliptic Envelopes **Input**: SHM vector ψ **Data**: Logistic regression model *M*, entropy window *E* of size *W*, significance factor *s* 
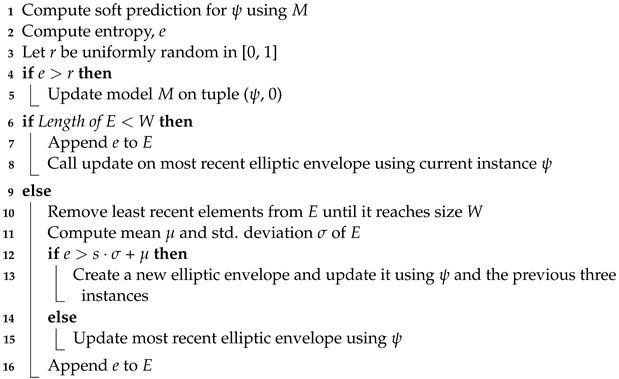


## 6. Assessing Performance

Denote by *D* and *H* the set of damaged and healthy instances and by $S_{f}$ the instances which were used in updating the model, which are denoted as *f*. $S_{i}$ refers to the set of all instances in the *i*th scenario of the bridge. The first criteria, shown in Equation (17), is the test accuracy (TA), which is the percentage of damage instances, unseen during training, which were correctly classified as damage.

(17) $$\mathrm{Test}\;\mathrm{accuracy}\;=\frac{\sum_{i\in D\cap \bar{S_{f}}}1_{f(x_{i})=-1}}{|D\cap \bar{S_{f}}|}$$

I use the test accuracy to determine how well each unsupervised model generalizes to the damage states. The second criteria is the false positive rate (FPR) of Equation (18).

(18) $$FPR=\frac{\sum_{i\in H}1_{f(x_{i}=-1)}}{\left|H\right|}$$

Finally, we use the cross-validation score to improve the precision of the model. The cross-validation score is computed as in Equation (19).

(19) $$\mathrm{CV}-\mathrm{score}=\frac{1}{|\left\{S_{i}\right\}{|}_{S_{i}\subset H,i\neq 1}}\sum_{j\neq 1,S_{j}\subset H}\frac{\sum_{i\in S_{j}}1_{f(x_{i})=1}}{|S_{j}|}$$

Cross-validation is used to find the optimal parameters of each model, thereby maximizing the precision of the model on healthy instances. Therefore, despite not being particularly designed to optimize damage detection, cross-validation is fundamental as it contributes to reducing the cost of inspection. Due to the tradeoff between the FPR and damage-case accuracy, it is important that these be defined for each campaign in proportion to the cost of inspection of a bridge as well as continued safe operation of the bridge.

## 7. Results

In this study on the Z24 bridge, we used 2.7 min for each epoch as it resulted in an even distribution of epochs among scenarios as well as an increase in efficiency of the computation of frequency-domain representations such as the FFT. We conducted a preliminary test to see if it is possible to reduce the 2.70 min epochs used in this study to 1.35 min (halving the original duration from 16,384 to 8192 samples) without reducing the accuracy. We found that it is possible; however, the features, which were needed to achieve similar performance to that of the longer epochs, increased by approximately two-fold. Likewise, the required PCA components needed also increased to around five or six components. Hence, the decrease in Fourier transform resolution caused the estimates to become more noisy, thereby necessitating more features for system identification. As the Z24 spanned 60 m, to collect a minute of data for use in crowdsourced sensing [28], a vehicle would need to traverse it at 3.7 kph, which is not realistic.

**Figure 15:**
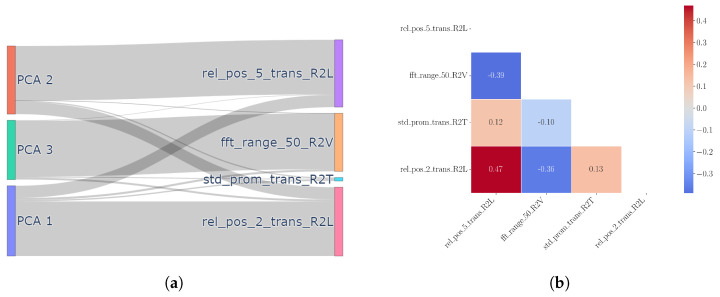
XGBoost demonstrated superior disentanglement with regard to PCA components. All PCA components approximately have a one-to-one mapping to original features. (**a**) Weight magnitudes of PCA eigenvectors, used in ψ, on the original features, denoted ϕ. (**b**) Correlation matrix of the original features.

**Figure 16:**
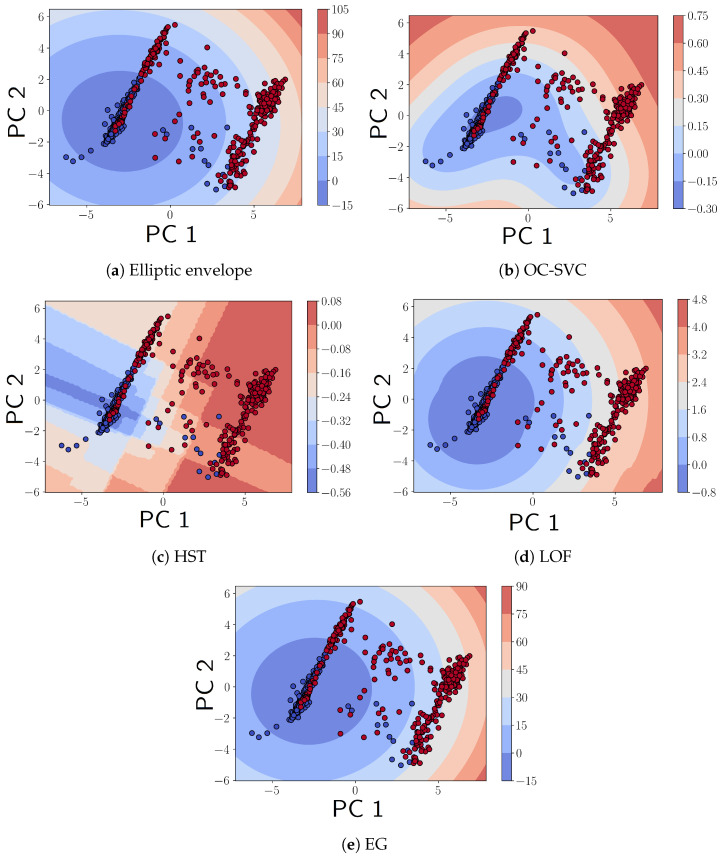
Projection of the decision surface for (**a**) elliptic envelope, (**b**) one-class SVC, (**c**) half-space trees, (**d**) local outlier factor, and (**e**) the last elliptic envelope formed in entropy-guided envelopes, respectively, onto two-dimensional PCA space. Negative regions are regions where the model outputs no anomaly, while positive regions are anomalous.

We begin by analyzing the feature extraction. With regard to feature extraction, XGBoost produced the PCA components that had the most disentanglement; i.e., each PCA component was almost uniquely mapped to a feature with very minor weights from other features. This is shown in Figure 15a. This can be intuitively understood given that XGBoost relies on the feature importance produced by impurity measures that divide original feature space quasi-orthogonally rather than distance-based measures like logistic regression or Euclidean distance (KNN). Hence, it is also possible to detect the online anomaly directly on the original features rather than on the PCA components. The decision boundaries for the different OCC methods [Section 5.1, Section 5.2, Section 5.3, Section 5.4 and Section 5.5] are shown in Figure 16.

**Figure 17:**
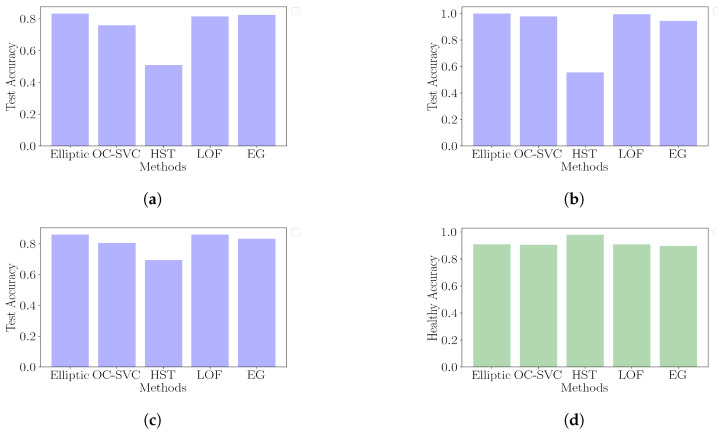
Accuracy categorized by damage type. (**a**) Accuracy for concrete damage detection. (**b**) Accuracy for tendons and anchor damage detection. (**c**) Accuracy for landslide damage detection. (**d**) Accuracy for healthy domain (1-FPR).

This study highlighted various anomaly detection methods applied to structural health monitoring, focusing on their ability to distinguish between normal and damaged states of infrastructure, such as bridges. Additionally, most methods presented similar accuracies for different types of damage as shown in Figure 17, except for half-space trees, which had better accuracy for healthy states than for damage. The reason half-space trees offer this performance is due to the large half-splits in the feature space, which produced a decision boundary with a relaxed fit around the support instances. The elliptic envelope method had constant time fitting and prediction and minimal memory requirements. We used a threshold based on a confidence level of 0.95 to identify anomalies. Despite its effectiveness in detecting significant damage, it struggled with early-stage damage detection due to minor deviations from the norm and adapting to later modes of healthy points, which were closer to the damage points than the early healthy points.

Despite its higher computational complexity, the incremental OC-SVC showed inferior performance compared to the elliptic envelope with a higher false positive rate and lower test accuracy, as shown in Table 4. Its complex decision surface hinders its ability to generalize across different undamaged scenarios, highlighting its impracticality for real-world applications. This method’s decision boundary overfitting indicates a difficulty in accommodating the diverse characteristics of structural health data, especially when non-linear separability is assumed through the use of the RBF kernel.

Half-space trees offer a notable improvement in reducing false positives with the highest ratio of damage test accuracy to false positive rate. However, the relatively low test accuracy is a hindrance to their applicability for critical infrastructure monitoring. The method’s sensitivity to tree depth in relation to support volume as well as the online min–max scaling of features are a limitation to its wide-scale applicability.

The local outlier factor method produced a decision boundary similar to the elliptic envelope, as shown in Figure 16a. A point worthy of study, which was not considered here, is the deviation of consecutive instances with respect to the damage class. Given the high performance of neighbor-based methods and that damage instances are on average farther apart, it is possible to compute consecutive deviations and add them as an extra feature to the points.

Meanwhile, entropy-guided envelopes offered a dynamic approach to model adaptation, leveraging entropy spikes to reset the model in response to significant changes in data distribution. The entropy slowly (though irregularly) decreased for healthy samples (the first 288 samples) and then peaked during the start of the damage. These irregular spikes during the healthy system can be smoothed by analyzing incoming points in batch sizes greater than one.

## 8. Conclusions

**Figure 18:**
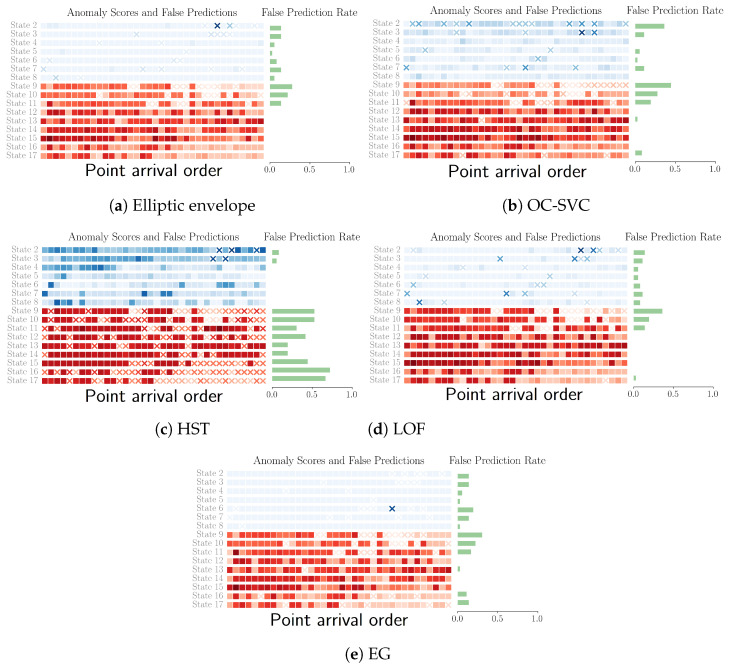
Anomaly scores for the different OCC techniques. Color intensity correlates with anomaly score. False predictions are shown with a cross. The blue region contains the points seen during training that are predicted as healthy/non-anomalous, and red corresponds to the damage evaluation set. For states, refer to Table 1.

Our work demonstrated the utility of simple features derived from the PSD graphs without the use of computationally-heavy modal analysis. With regard to the distribution of misclassification in Figure 18, all the methods presented the same pattern of misclassification, suggesting that the limitation in accuracy is mainly due to the features and not the predictive model. Thus, we should expect bagging over different feature sets to perform better than bagging over different models that use the same features.

Entropy-guided elliptic envelopes performed poorly due to the fact that when a new envelope is formed, the false positive rate is very high, yielding a criterion score of 0.166. Additionally, the local outlier factor had a score of 2.85 followed by incremental SVC with 2.72 and then the elliptic envelope and half-space trees with 2.36 and 1.92, respectively.

(20) $$J_{f}=\frac{{\left(m_{D,f}-m_{H,f}\right)}^{2}}{s_{D,f}^{2}+s_{H,f}^{2}}$$

**Figure 19:**
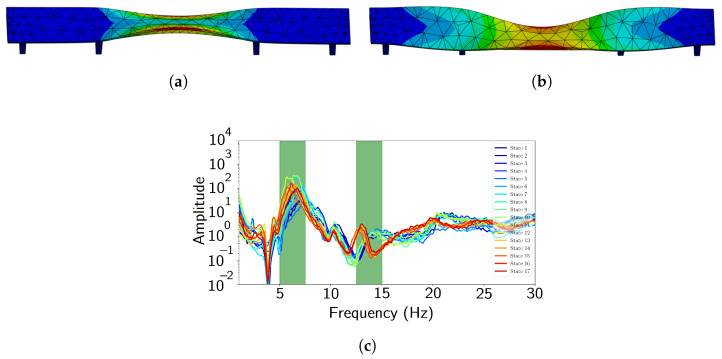
Z24 model in ANSYS 2023 R1. The outermost piers are embedded within the ground elevations and hence are not visible. Two of the four features used, listed in Figure 7, are shown in the bottom right and bottom left figures. (**a**) Second mode shape of the Z24 bridge. (**b**) Fourth mode shape of the Z24 bridge. (**c**) Transmittance of R2L relative to R1V. In green are the windows from which we extract the relative position of the peaks. These constitute the first and last features of Figure 7.

The graphs shown in Figure 19 reflect the natural interpretation of the features: significant windows overlap with locations of natural frequencies. Although not formally part of the performance criterion, it may be useful to compare the methods in terms of the Fisher criterion of Equation (20) in order to assess the quality of the separation between anomaly scores for healthy and damage data [27]. This demonstrates that similarity in terms of decision surface does not necessarily correlate with the quality of separation of anomaly scores.

## 9. Future Work

There are several extensions possible to this study. Most box girder bridges can be defined parametrically using numerical inputs as well as their type. Based on these inputs, a bridge can then be procedurally generated. Thus, the cost of human modeling can be alleviated by having a program which generates a bridge. Once the automated modeling is complete, upon input, the bridge description has a deep neural network (DNN) that meta-learns a priori on the hyperparameters that would result from performing cross-validation on healthy scenarios. This extension would only be possible using programmable bridge models as the DNN would require a large number of bridge models to train [29,30].

Another potential extension involves a comparative performance analysis of modal analysis against frequency-based feature extraction methods, such as those explored in this study, to environmental and meteorological variables [31]. Specifically, examining the sensitivity of extracted features to variations in external conditions and the impact of window selection in feature extraction is crucial. For example, a deviation from the natural frequencies could significantly influence the results. As shown in Figure 19, many of the windows are not centered precisely on the modal frequencies, highlighting the need to determine how off-center a window can be before a significant drop in accuracy occurs. This analysis could build upon existing work, which focuses on temperature and wind speed impacts on modal parameters [32,33].

Additionally, integrating machine learning advancements can enhance feature extraction techniques. This study used a model-free approach with supervised feature extraction, but future work could leverage convolutional neural networks (CNNs) trained on spectrograms of structural health monitoring (SHM) tasks with labeled datasets, such as the LUMO dataset [34] or data from the SHMnet framework [35]. Using a pretrained CNN for feature extraction from other SHM tasks (source datasets), such as the LUMO dataset, may obviate the need for supervised feature extraction on the target dataset, which is a hindrance to the real-world applicability of this approach [34,36]. Incorporating a high-fidelity numerical model could help identify the most relevant frequency-based features and guide the analysis process [37].

Exploring the fusion of SHM data with data-driven models that incorporate uncertainty quantification could also provide more robust monitoring solutions [38,39]. Finally, employing transfer learning strategies between different types of bridges or structural elements could significantly reduce the need for extensive training data [40,41,42].

## Figures and Tables

**Table 1:** List of bridge states. The first eight states are considered healthy.

Index	Date (1998)	Scenario		
State 1	4 August	No damage	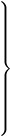	healthy
State 2	9 August	No damage (Koppigen pier installation)		
State 3	10 August	Settlement of pier (20 mm)		
State 4	12 August	Settlement of pier (40 mm)		
State 5	17 August	Settlement of pier (80 mm)		
State 6	18 August	Settlement of pier (95 mm)		
State 7	19 August	Tilt of foundation		
State 8	20 August	New reference (pier installed)		
State 9	25 August	Spalling of concrete ($12\;m^{2}$)		
State 10	26 August	Spalling of concrete ($24\;m^{2}$)		
State 11	27 August	Landslide (1 m)		
State 12	31 August	Failure of concrete hinges		
State 13	2 September	Failure of 2 anchor heads		
State 14	3 September	Failure of 4 anchor heads		
State 15	7 September	Failure of 54 tendon wires		
State 16	8 September	Failure of 100 tendon wires		
State 17	9 September	Failure of 154 tendon wires		

**Table 2:** Features extracted from detrended and band-pass filtered signal.

Feature	Length
Descriptive statistics (moments)	8
Lengths, repetitions	9
Autocorrelations, complexity metrics	6
Statistical combinations	9
Moving average features	6
Descriptive statistics on frequency domain	6
Cumulative statistics on PSD and FT	4
Total	48

**Table 3:** Features extracted from PSD and transmittance.

Feature	Length
Descriptive statistics ($\mu_{X[f]},count_{\mathrm{peaks}},\sigma_{\mathrm{height}}\cdots$)	10
Piecewise polynomial regression coefficients (deg=3)	24
Largest peak indices	4
Sharpest peak positions	4
Sharpest peak heights	4
Peak height in all 2.5 Hz windows, offset w.r.t to window	24
Spectral centroid	4
Total	74

**Table 4:** Performance comparison of all five OCC techniques.

	Test Accuracy	FPR	CV-Score
Elliptic [Section 5.1]	0.93	0.08	0.92
OC-SVC [Section 5.2]	0.89	0.10	0.97
HST [Section 5.3]	0.56	0.02	0.99
LOF [Section 5.4]	0.92	0.09	0.93
EG [Section 5.5]	0.89	0.10	0.90

## Data Availability

The Z24 bridge benchmark dataset is available upon request at the following address: https://bwk.kuleuven.be/bwm/z24, accessed on 1 August 2023.

## Abbreviations

The following abbreviations are used in this manuscript:

SHM	structural health monitoring
FT	Fourier transform
PSD	power spectral density
t-SNE	t-stochastic neighborhood embedding
NI	node impurity
KNN	K-nearest neighbors
LOF	local outlier factor
LRD	local reachability density
EG	entropy-guided envelopes
SVC	support vector classifier
RBF	radial basis function
HST	half-space trees
DNN	deep neural network
PCA	principal component analysis
LUMO	Leibniz Universtity Test Structure for Monitoring

## Appendix A. Hyperparameter Values

### Appendix A.1. Feature Selection

**Table A1:** Hyperparameter grid used during feature selection.

XGBoost	Values
Learning rate	[0.05, 0.1, 1]
Maximum depth	[3, 5, 7]
Minimum child weight	[1, 3, 5]
Gamma	[0.1, 0.5, 1]
Subsample	[0.4, 0.7, 1]
Column sample for each tree	[0.3, 0.4, 0.5]
Estimator count	[35, 100, 140]
Logistic regression	Values
C	[0.1, 0.2, 0.4]
Penalty	L1
Solver	liblinear
SVC	Values
C	[0.1, 0.5, 1, 2]
Gamma	[‘auto’, ‘scale’]
Kernel	[‘linear’, ‘rbf’]
KNN	Values
Distance (p)	Manhattan (1) and Euclidean (2)
Neighbor count	[5, 10, 20, 40]
Relief	Values
Neighbor count	[2, 4, 8, 16]
Features	[4, 8, 16, 32, 64, 128]

### Appendix A.2. Online Anomaly Detection

**Table A2:** Hyperparameter grid used during anomaly detection.

Incremental SVC	Values
Nu	[0.01, 0.1, 0.2]
Buffer size	[9, 18, 36]
Retrain size	[10, 15, 20]
Half-space trees	Values
Window size	[15, 30]
Height	[3, 4]
Number of trees	[10, 50, 100]
Local outlier factor	Values
Window size	[9, 18, 36]
Neighbor count	[3, 6, 12, 24]
KNN	Values
Nu (logistic regression model)	[0.1, 0.3, 0.6]
Significance factor	[1, 2, 3]
Entropy window	[5, 10, 15, 20]
